# Linking common human diseases to their phenotypes; development of a resource for human phenomics

**DOI:** 10.1186/s13326-021-00249-x

**Published:** 2021-08-23

**Authors:** Şenay Kafkas, Sara Althubaiti, Georgios V. Gkoutos, Robert Hoehndorf, Paul N. Schofield

**Affiliations:** 1 Computational Bioscience Research Center (CBRC), Computer, Electrical, and Mathematical Sciences & Engineering Division, King Abdullah University of Science and Technology, 4700 KAUST, Thuwal, 23955 Saudi Arabia; 2 Health Data Research UK, Midlands site, Edgbaston, Birmingham, B15 2TT United Kingdom; 3 Institute of Cancer and Genomic Sciences, University of Birmingham, Edgbaston, Birmingham, B15 2TT United Kingdom; 4 Department of Physiology, Development & Neuroscience, University of Cambridge, Downing Street, Cambridge, CB2 3EG United Kingdom

**Keywords:** Disease–phenotype associations, Ontologies, Text mining, UK Biobank

## Abstract

**Background:**

In recent years a large volume of clinical genomics data has become available due to rapid advances in sequencing technologies. Efficient exploitation of this genomics data requires linkage to patient phenotype profiles. Current resources providing disease-phenotype associations are not comprehensive, and they often do not have broad coverage of the disease terminologies, particularly ICD-10, which is still the primary terminology used in clinical settings.

**Methods:**

We developed two approaches to gather disease-phenotype associations. First, we used a text mining method that utilizes semantic relations in phenotype ontologies, and applies statistical methods to extract associations between diseases in ICD-10 and phenotype ontology classes from the literature. Second, we developed a semi-automatic way to collect ICD-10–phenotype associations from existing resources containing known relationships.

**Results:**

We generated four datasets. Two of them are independent datasets linking diseases to their phenotypes based on text mining and semi-automatic strategies. The remaining two datasets are generated from these datasets and cover a subset of ICD-10 classes of common diseases contained in UK Biobank. We extensively validated our text mined and semi-automatically curated datasets by: comparing them against an expert-curated validation dataset containing disease–phenotype associations, measuring their similarity to disease–phenotype associations found in public databases, and assessing how well they could be used to recover gene–disease associations using phenotype similarity.

**Conclusion:**

We find that our text mining method can produce phenotype annotations of diseases that are correct but often too general to have significant information content, or too specific to accurately reflect the typical manifestations of the sporadic disease. On the other hand, the datasets generated from integrating multiple knowledgebases are more complete (i.e., cover more of the required phenotype annotations for a given disease). We make all data freely available at 10.5281/zenodo.4726713.

**Supplementary Information:**

The online version contains supplementary material available at (10.1186/s13326-021-00249-x).

## Background

The genomic revolution in medicine has been driven by the huge success of next generation sequencing and the availability of millions of genome or exome sequences [1, 2]. However, the utility of genomic sequence is determined firstly by our knowledge of the relationship between genomic variants and disease conditions or predispositions [3–5], and secondly by our knowledge of the relationship between protein function and sequence [6, 7]. To date, the application of this knowledge has been focused in two areas, rare diseases, generally Mendelian [8], and common or complex diseases [5, 9].

A significant breakthrough in rare disease diagnostics and candidate gene discovery was facilitated by the development of phenotype ontologies that capture the phenotypes associated with a disease entity. These are now available not only for human and most of the model organisms [10, 11], but also as unified and integrated phenotype ontologies [12, 13] where the equivalences and relationships between phenotypes in different species are captured. Use of these ontologies to establish phenotypic similarity between undiagnosed patients and known human disease entities, or model organism mutants, has provided useful new diagnostic support and discovery tools [14–16]. This work has benefited considerably from careful phenotypic characterization of the known rare recurrent and Mendelian diseases of which there are estimated to be about 7,000 [10, 17, 18]. Efforts to annotate complex and common diseases with their phenotypes have been limited by the scale of the task, with 14,400 distinct diagnoses and causes of death identified in ICD-10 [19] and 69,000 diagnosis codes in ICD-10-CM, of which many are phenotypic variants.

The advantage of creating a corpus of phenotype annotations to common diseases is that they can then be used to computationally search for phenotypic and genetic associations between phenotypes or diseases, for identification of new phenotypic subgroups, and for diagnostic support and facilitation of the analysis of electronic patient record data [20–23]. In recent years, evidence has accumulated to support a model in which different diseases have common underlying etiopathological mechanisms and shared phenotypes, or endophenotypes [24, 25]. The hypothesis that similarity between phenotypes reflects underlying biological modules of functionally related genes has been convincingly demonstrated for Mendelian disease but little work has been done for common and complex disease [26, 27]. An earlier study of common diseases [28] suggested that these diseases form modules related to Mendelian genes with similar phenotypes. Similarly, the phenotype study by Ghiassian et al. [24] took three selected endophenotypes (inflammation, thrombosis, and fibrosis) and genes annotated to these, to show that the genetic modules associated with each phenotype interacted together to generate inflammation.

We have previously used text mining to annotate the diseases covered by the Human Disease Ontology (DO) [29], which contains both common and rare diseases, and demonstrated that phenotypically closely related diseases are linked at the level of underlying etiology and align with existing nosology [30]. However, this set of annotations was limited at the time to those diseases in DO and used only literature-derived associations. Many disease-phenotype pairs have been gathered from human genetic studies and animal model experiments and are now available from large-scale public resources [31, 32]; one aim of the current study was to leverage these curated public resources alongside a more comprehensive text mining effort. However, these resources are far from being complete, and few phenotypes are linked to terms found in ICD-10 [33], which is the primary disease terminology used in clinical practice. A large number of disease–phenotype associations are still latent in the literature and require automated methods to extract.

Here, we focus on the diseases in ICD-10 and link them to their relevant phenotypes from the Human Phenotype (HPO) and Mammalian Phenotype ontologies (MP) [34, 35]. We present two approaches to gather disease-phenotype associations. One of them is text mining from the literature and the other one is semi-automatic harvesting from publicly available curated resources. To extract the disease-phenotype associations from text, we utilize the semantics of the PhenomeNET ontology to increase the coverage of annotations that are not explicitly mentioned [12] in text, and apply a statistical approach to find significant associations between a disease and sets of phenotypes. We evaluate our text mining predictions against the known disease–phenotype associations from the HPO database [31]. Furthermore, we demonstrate the utility of the generated datasets in predicting gene–disease associations from Mouse Genome Informatics (MGI) [32] based on phenotype similarity. We provide all of the datasets of disease–phenotype associations at 10.5281/zenodo.4726713.

## Materials and methods

### Resources used

#### Diseases

We have built a semantic disease resource from the Unified Medical Language System (UMLS) [36] and use it for disease–phenotype association extraction. We use UMLS due to its completeness, but also its extensively validated mappings to other terminologies, specifically ICD-10 and HPO, which are not available elsewhere. As one of our main aims is to facilitate the use of data in Electronic Health Records, we focus here on diseases included in the ICD-10. However, it is straightforward to map our resource onto other known disease resources such as DO [29] and Mondo Disease Ontology [37] through the ICD mappings in them.

To generate our disease resource, we first parsed UMLS data (from the file MRCONSO.RRF in UMLS, downloaded on 04/11/2019) and gathered all the disease concepts along with their labels, definitions and sub-class relations which were mapped to any of the classes in ICD-10, SNOMED CT, HPO, or Online Mendelian Inheritance in Man (OMIM) [38]. We then represent the resulting integrated resources using the Web Ontology Language (OWL) [39] where we assert rdfs:subClassOf between two classes that are sub-classes in UMLS, and rdfs:label properties based on all the collected labels and definitions. Although our main focus was ICD-10 diseases, we included disease classes represented in SNOMED, HPO, and OMIM as well in the generation of this resource so as to benefit from their ontological structure and maintain an asserted hierarchy among the disease concepts. This semantic disease resource covers a total of 1,535,927 disease labels from 519,735 disease concepts. We used this data to identify disease mentions (names, synonyms and acronyms) in text.

#### Phenotypes

We used two phenotype ontologies, HPO and MP, to identify phenotypes in text. Both ontologies contain classes that are relevant to humans, so to cover the complete phenotype profile of a given disease as completely as possible we used MP in addition to HPO. We used only the subclasses of the *Phenotypic abnormality* branch of HPO (14,749 HPO classes in total)to generate the text-mined dataset as this branch covers phenotypes that can be readily associated with diseases. For the semi-automatically generated dataset, we considered all of the HPO classes as the data is seeded from curated annotations. We used primary and alternative class labels along with synonyms in text matching. We used the PhenomeNET [40] ontology, which includes the phenotypes from HPO and MP, to generate embeddings for diseases and genes. Briefly, PhenomeNET is developed by transforming phenotype ontologies into a formal representation, combining phenotype ontologies with anatomy ontologies, and applying a measure of semantic similarity to construct a cross-species phenotype network.

#### Known disease-phenotype, gene-phenotype and gene-disease associations

We gathered the known disease–phenotype associations from UMLS [36], the HPO database [31] on 12/10/2020 and Wikidata [41] on 13/09/2020. We gathered the known mouse gene–phenotype [42] as well as gene–disease associations [43] from MGI [32] on 15/03/2021.

#### UK biobank

We generated a list of 2,106 common diseases from the UK Biobank identified by their ICD-10 codes. To generate this list, we considered only ICD-10 codes that have 100 or more patients in UK Biobank, as identified through the main or secondary diagnosis fields.

### Generating disease-phenotype associations

**Fig. 1:**
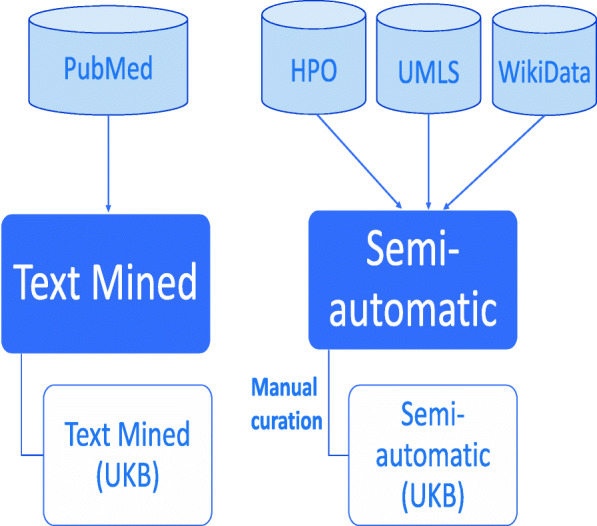
Overview of generating disease-phenotype associations. We used two methods, text mining and a semi-automatic way to generate two independent datasets. We further generated subsets of these datasets covering the phenotype associations of common diseases from UK Biobank. To generate the semi-automatic (UKB) dataset, we manually curated the selected diseases

In this study, we developed two methods; one of them is a text-mining based method and the other one is a semi-automatic way to gather asserted disease–phenotype associations from public data resources. By using these two methods, we generated a total of four datasets in this study. Figure 1 depicts an overview of the processes applied and the datasets generated. First, we generated two independent datasets; one by text mining disease–phenotype associations from PubMed abstracts (this dataset is labeled “Text Mined”) and an another one by semi-automatically gathering the known disease-phenotype associations from multiple resources (labeled “Semi-automatic”).

Linking genotype and phenotype can be extremely helpful in DNA sequence analysis for revealing causative variants. Therefore, we selected the disease–phenotype associations linked to common diseases in the UK Biobank from these two datasets. In the case of the Text Mined dataset, we retrieved all of the selected associations into the data subset we call “Text Mined (UKB)”. In the case of the Semi-automatic dataset, we further applied expert manual curation on the selected associations and generated the “Semi-automatic (UKB)” dataset.

#### Text mining disease-phenotype associations

**Fig. 2:**
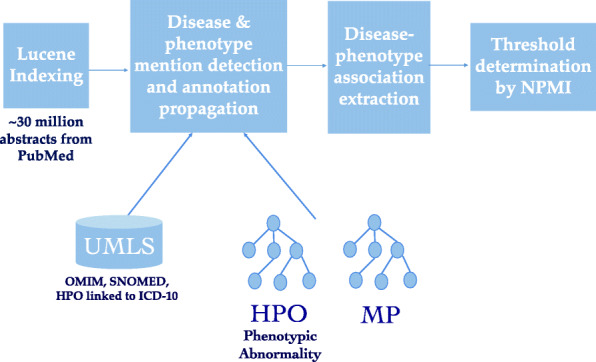
Overview of text mining disease-phenotype associations. Disease labels are gathered from OMIM, SNOMED and HPO records which are linked to ICD-10 in UMLS. Phenotype labels are gathered from HPO and MP

Figure 2 depicts the overview of text mining of disease-phenotype associations. To extract disease–phenotype associations, we first indexed approximately 30 million PubMed abstracts downloaded on 22/09/2019 from ftp://ftp.ncbi.nlm.nih.gov/pubmed/baseline using Apache Lucene [44]. Secondly, we identified the abstract level occurrence and co-occurrence of each disease–phenotype pair. We then propagated the co-occurrence statistics by using the semantics in PhenomeNET (i.e., if *C* is a subclass of *D* in the PhenomeNET ontology, every mention of *C* in an abstract is also considered a mention of *D*). and calculated the normalized pointwise mutual information (NPMI) [45] to measure the strength of the association.

NPMI is a measure of collocation of two terms. As the disease and phenotype concepts are represented by ontology classes, we reformulated the NPMI to measure the collocation between two classes. First, we identify the set of labels and synonyms associated with every class; *Labels*(*C*) denotes the set of labels and synonyms of *C*. We then define *Terms*(*C*) as the set of all terms that can be used to refer to *C*: $Terms(C) := \{ x | x \in Labels(S) \land S \sqsubseteq C \}$.

We calculated the NPMI between classes *C* and *D* as

1 $$ npmi(C,D) = \frac{\log{\frac{n_{C,D}\cdot n_{tot}}{n_{C} \cdot n_{D}}}}{-\log{\frac{n_{C,D}}{n_{tot}}}}  $$

where *n*_*tot*_ is the total number of abstracts in our corpus in which at least one disease and one phenotype name co-occur, *n*_*C*,*D*_ is the number of abstracts in which both a term from *Terms*(*C*) and a term from *Terms*(*D*) co-occur, *n*_*C*_ is the number of abstracts in which a term from *Terms*(*C*) occurs, and *n*_*D*_ is the number of abstracts in which a term from *Terms*(*D*) occurs.

#### Gathering disease-phenotype associations from public data sources

**Fig. 3:**
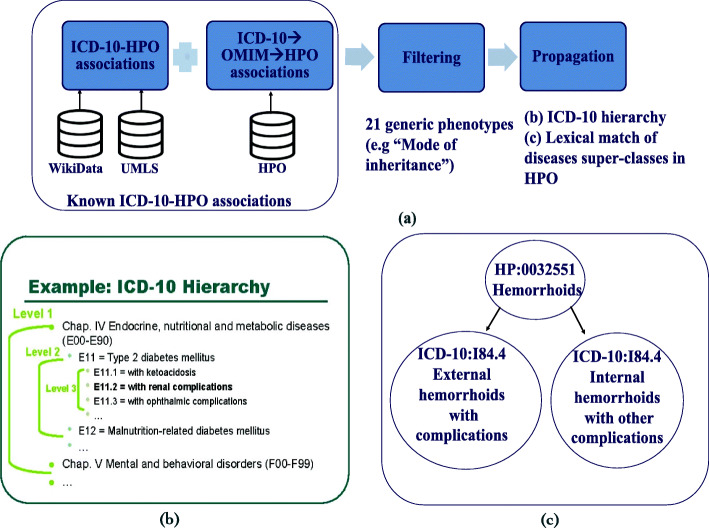
Overview of semi-automatic gathering known disease-phenotype associations. (a) Collecting known ICD-10-HPO associations from WikiData, UMLS and HPO, filtering associations with generic phenotypes and propagation based on ICD-10 hierarchy and lexical match of disease super-classes in HPO. (b) A sample ICD-10 Hierarchy. (c) A sample lexical match of disease super-class in HPO

We generated the Semi-automatic dataset of disease–phenotype associations based on gathering known associations from UMLS, Wikidata, and the HPO database, and then propagating them based on the superclass relations defined in ICD-10 and lexical match of superclasses of diseases in the HPO dataset (see Fig. 3). To generate this dataset, first, we gathered the known disease–phenotype associations from UMLS and Wikidata. We collected a total number of 2,340 ICD-10–HPO direct mappings for 2,268 distinct diseases from UMLS. We used SPARQL queries and retrieved a total number of 2,029 distinct associations for 404 distinct diseases (mapped to ICD-10) with their symptoms and secondary effects (mapped to HPO) from Wikidata; the SPARQL queries we used are available as Supplementary Materials. Second, for capturing the known disease–phenotype associations from the HPO database, we applied an additional disease identifier conversion step. The HPO database contains disease–phenotype associations where the diseases are mapped to OMIM and the phenotypes are mapped to HPO. To include these associations, we mapped the OMIM diseases to their ICD-10 codes by using the ICD-10–OMIM mappings from UMLS and Wikidata. We gathered the mappings from Wikidata using another SPARQL query (see). We extracted 303 and 5,747 ICD-10–OMIM mappings from UMLS and Wikidata, respectively. Merging these two resources, we obtained a total of 5,845 distinct ICD-10–OMIM mappings where 1,447 distinct ICD-10 codes are mapped to their corresponding OMIM identifiers. Altogether, utilizing the obtained ICD-10–OMIM mappings, we gathered a total number of 41,529 ICD-10–HPO associations for 1,366 distinct diseases from the HPO database. Third, we filtered out the associations involving 21 generic, not informative phenotypes which were manually identified by an expert from the dataset (e.g., HP:0000005*Mode of inheritance*, HP:0000006*Autosomal dominant inheritance*, HP:0012824*Severity*, HP:0025285*Aggravated by*, HP:0012834 Right). Fourth, we propagated the known annotations based on the superclass relations in ICD-10 coding system hierarchy. For example, phenotypes linked to (ICD-10:G30) *Alzheimer’s disease* are propagated to all 4 of its sub-classes (ICD-10:G30.0 Alzheimer’s disease with early onset, ICD-10:G30.1 Alzheimer’s disease with late onset, ICD-10:G30.8*Other Alzheimer’s disease*, ICD-10:G30.9*Alzheimer’s disease, unspecified*). We also propagated annotations to the diseases from their superclasses that we find by lexical match in the HPO database. For example, we linked ICD-10:I84.4, *External hemorrhoids with complications* to *Hemorrhoids* (HP:0032551) (see Fig. 3c).

#### Manual curation of “Semi-automatic” disease–phenotype associations

Linking genotype and phenotype is important for understanding the underlying mechanisms of genomic disorders. Therefore, we selected the phenotype associations of the common diseases in UK Biobank from this dataset and manually curated them. We named this curated dataset “Semi-automatic (UKB)” and released it as an additional resource. To generate this subset of data, we first identified and prioritized a total of 2,106 diseases which are common, defined as those for which at least 100 individuals in UK Biobank have either a primary or secondary diagnosis. Second, we retrieved the known and propagated associations of these 2,106 diseases. Third, we manually added the phenotypes for the diseases for which we could not find any associations after applying the second step. Missing ICD-10–HPO annotations were provided by expert curation and reference to the literature.

Clinical presentation was checked initially using expert knowledge supported by standard texts [46, 47] and then examined in further depth using recent literature reviews and papers. Examination of the HPO classes annotated to diseases in this semi-automated dataset revealed several broad curation strategies for annotation taken by each contributing dataset; UMLS, Wikidata, and HPO database. Each type of annotation was not limited to one data source (for example direct mapping was found in all contributing datasets), and reflects the various strategies and individual pragmatic decisions adopted by the contributing resources. This is a compromise in comparison with a priori expert annotation, but our overall validation suggests that it does not compromise the utility of our datasets. Several examples are shown below:

1. *Direct mapping* In some cases, HPO classes reflect a simple mapping from ICD-10 to HPO; for example ICD-10:N20.0*Calculus of kidney* is annotated with HP:0000121 Nephrocalcinosis.
2. *High level mapping*ICD-10:H40.1, Primary open-angle glaucoma is annotated with HP:0000478, *Abnormality of the eye*.
3. *Symptom manifestation*ICD-10:C91.1, Chronic lymphocytic leukaemia is annotated with HP:0040088, *Abnormal lymphocyte count*
4. *Associated phenotypes including etiological predication, and closely related diseases or phenotypes*ICD-10:E21.0, *Primary hyperparathyroidism* is annotated with HP:0011769, *Ectopic parathyroid*; similarly, ICD-10:D75.2, *Essential thrombocytosis*, is annotated with HP:0011974, *Myelofibrosis*, a closely linked disorder [48].

Each method of assigning annotations used by the contributing resources had, to a greater or lesser degree, a bias towards one or more of these patterns (see below). While restricting annotations simply to signs and symptoms would have met the aim of disaggregating diseases into their constituent phenotypes, much information would have been lost and in fact HPO itself includes annotations of all of these types to advantage. We show below that using all of the methods of annotation resulted in a much better predictive outcome on the disease-gene validation task, justifying the inclusion of all of these types in annotation. The possible bias introduced by annotation density and the level of annotation to superclasses is discussed below.

In the last step, the generated ICD-10–HPO associations for 2106 ICD-10 codes were assessed by a biomedical expert for inappropriate and incorrect HPO annotations (false positives) and removed. The final number of common ICD-10 codes that could be linked to their phenotypes is 1,995.

### Measuring phenotypic similarities of genes and diseases

We used OWL2Vec* [49] to generate embeddings for entities based on their associations with phenotypes. OWL2Vec* is a method to generate embeddings for classes in OWL ontologies. OWL2Vec* converts an ontology into a graph based on syntactic patterns represented in the ontology axioms; in the graph, nodes correspond to either classes or individuals in the ontology, and edges correspond to axiom patterns. OWL2Vec* then applies a graph embedding method based on random walks to nodes in this graph; it explores the node neighborhood through iterated random walks with a subtree kernel and uses Word2Vec [50] to embed nodes within a vector space.

For each disease, we generated two ontology embeddings, one based on the text mined phenotype profile and the other on the phenotype profile from the HPO database. We used the default parameter settings of the OWL2Vec* implementation: vector size 100, window size 5, minimum occurrence count of 1, skip-gram (sg) model, random walk with depth (number of walk) of 3.

We measured the similarity between the ontology embeddings using cosine similarity:

2 $$ \text{sim}\left(v_{1}, v_{2}\right)=\frac{v_{1} \cdot v_{2}}{\left\|v_{1}\right\|\left\|v_{2}\right\|}  $$

where *v*_1_ and *v*_2_ are two vectors representing two given entities.

## Results

### Disease–phenotype datasets

We generated a total of four datasets covering disease–phenotype associations (see Fig. 1) by using text mining and semi-automatic collection of associations from public data resources. The “Text Mined” and “Semi-automated” datasets contain all of the text mined and semi-automatically gathered ICD-10-phenotype associations respectively. “Text Mined (UKB)” is the subset of “Text Mined” which covers the associations of only common diseases found in UK Biobank. On the other hand, “Semi-automated (UKB)” covers further manually curated known associations of these common diseases. Table 1 presents the distribution of the associations in the generated datasets based on their provenance. Our aim is specifically to associate common diseases in ICD-10 with phenotypes so that we can map datasets using ICD-10 to phenotypes.

**Table 1:** Distribution of disease-phenotype associations in the generated datasets by provenance

Provenance	Text Mined	Text Mined (UKB)	Semi-automatic	Semi-automatic (UKB)
PubMed	2,755,333	985,511	-	-
Wikidata	-	-	1,838	295
HPO (through OMIM–ICD-10 from Wikidata)	-	-	32,323	3,914
HPO (through OMIM–ICD-10 from UMLS)	-	-	2,362	423
UMLS	-	-	1,287	541
Expert curation	-	-	-	433
Propagation(ICD-10)	-	-	10,201	1,214
Propagation(HPO)	-	-	9660	756
TOTAL	2,755,333	985,511	57,671	7,576

UKB denotes the subset covering common diseases only from UK Biobank

The Text Mined dataset covers a total of 2,755,333 positive disease–phenotype associations (NPMI score > 0) between 13,610 distinct phenotype classes (from either MP or HP) and 6,263 distinct diseases (from ICD-10) from the literature. A total of 985,511 out of 2,755,333 disease–phenotype annotations can be linked to 1,557 of 2,106 common ICD-10 codes (Text Mined (UKB)). For the remaining 549 diseases, we could not find any positive association from the literature based on our approach.

The Semi-automatic dataset covers a total of 57,671 ICD-10–HPO associations among 7,610 distinct ICD-10 classes and 6,741 distinct phenotypes obtained by integrating a number of manually curated datasets (see Table 1). Out of the 57,671 ICD-10–HPO associations, we gathered the majority of the associations (37,810 out of 57,671 associations, linked to 4,207 of the 7,610 ICD-10 classes) through resources covering rare or common diseases. We obtained a total of 1,838 association from Wikidata, 32,323 associations from the HPO database through OMIM–ICD-10 links from Wikidata and 2,362 through OMIM–ICD-10 links from UMLS; we also obtained 1,287 associations directly from UMLS. We gathered the remaining 19,861 associations (linked to 3,403 of the 7,610 ICD-10 classes) by propagating phenotype annotations of diseases from their subclasses in the ICD-10 hierarchy. We obtained 10,201 out of 19,861 associations by propagating phenotypes from their superclass based on the ICD-10 hierarchy; we obtained the remaining 9,660 out of 19,861 associations by lexical match between the superclass labels and the phenotype labels in HPO.

We sub-selected 2,106 distinct ICD-10 diseases from the Semi-automatic dataset covering all the common ICD-10 codes within UK Biobank. We curated their phenotype associations manually and filtered out the false positives. This curated dataset (Semi-automatic (UKB)) contains a total of 7,576 disease–phenotype associations gathered in a semi-automated way (see Materials and Methods) between 1,995 (of 2,106) common ICD-10 diseases and 2,757 distinct phenotypes linked to HPO. We gathered the majority of phenotype associations (4,337 out of 7,576 associations) for 334 distinct ICD-10 codes from HPO through ICD-10–OMIM links in either Wikidata (3,914/4,337 pairs) or UMLS (423/4,337 pairs). We gathered 541/7,576 associations linked to 473 distinct ICD-10 codes through direct mappings of ICD-10 and HPO in UMLS. We gathered 295/7,576 associations for 43 distinct ICD-10 codes from Wikidata. We generated 1,214/7,576 associations for 335 distinct ICD-10 codes by propagating phenotypes from their superclass based on the ICD-10 hierarchy.

We manually curated 433/7,576 disease–phenotype associations for 433 ICD-10 codes. We generated a total of 756/7576 associations linked to 483 ICD-10 codes by propagating phenotypes from their superclasses when we found a lexical match between the superclass labels and the phenotype labels in HPO.

### Phenotypic similarity of text mined and known associations

We measured the semantic similarity between our text mined and the known phenotypes of the diseases. There are 296 diseases in our dataset that are contained both in ICD-10 and OMIM and for which we can obtain phenotype associations both from our text mining approach and from curated data in the HPO database. We measured the semantic similarity between the phenotype profiles of a given disease by using cosine similarity between the ontology embeddings of the disease’s phenotype profiles generated through OWL2Vec* [49].

**Fig. 4:**
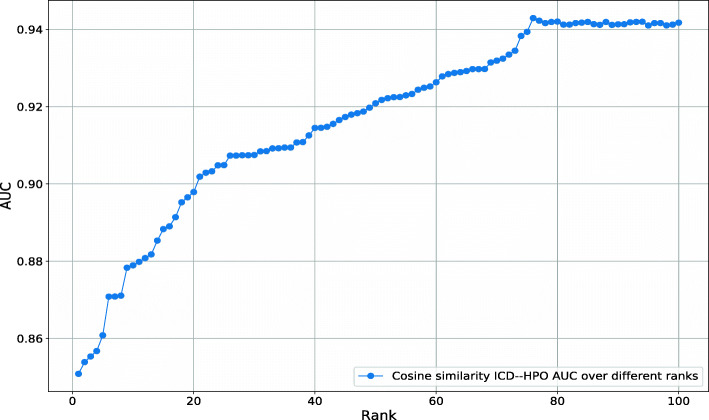
AUC values obtained for the phenotypic similarity of text-mined and known diseases from HPO at different NPMI ranks

Our Text Mined dataset consists of disease–phenotype associations and each association has a score that determines the association strength. Among the diseases in our dataset, between 1 and 2,592 phenotypes are positively associated. We assume that not all positive associations may be relevant but only the stronger associations provide useful information about a disease. We test this hypothesis by ranking phenotypes for each disease by their association (NPMI) score. We then include phenotypes in a disease–phenotype profile using varying thresholds for the number of phenotypes to include (based on the association score). To determine a threshold that yields a phenotype profile similar to manually curated ones, we compare the semantic similarity of the thresholded phenotype profiles to the manually curated profiles for the same disease; we evaluate the similarly using receiver operating characteristic (ROC) curves [51]. We find that a threshold of 76 phenotypes results in maximal similarity to the manually curated disease–phenotype associations (ROCAUC 0.95). Figure 4 shows the results of our experiment.

### Predicting gene-disease associations

We further evaluated whether our Text Mined and Semi-automatic (UKB) datasets are useful in identifying gene–disease associations based on phenotype similarity. We found 53 diseases in ICD-10 that can be mapped directly to OMIM and are also present in our Text Mined and Semi-automatic (UKB) datasets. These 53 diseases are associated with 216 genes in our gene–disease dataset gathered from MGI.

**Fig. 5:**
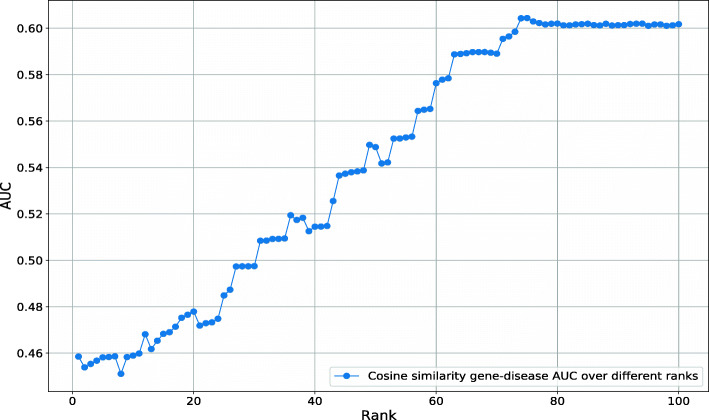
AUC values obtained for the phenotypic similarity of text-mined diseases and known genes from MGI at different NPMI ranks

Utilizing the text mined disease-phenotype associations with their association score, we followed a similar procedure as before and rank phenotypes for each disease based on their association score and vary the rank as threshold parameter. We then compared these phenotype profiles to phenotypes resulting from loss of function mouse models using the cosine similarity between their ontology embeddings, and evaluated how well this method recovers known gene–disease associations. Figure 5 shows the resulting ROCAUC at different NPMI ranks. We find the maximal ROCAUC value at rank 74 (ROCAUC 0.62).

**Fig. 6:**
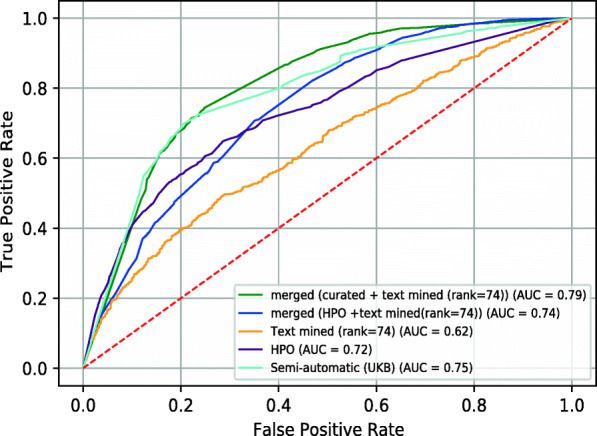
Comparison of ROC curves for predicting gene–disease associations using cosine similarity

We further used different datasets to find gene–disease associations through phenotype similarity: our Text Mined dataset with a threshold of 74 per disease; our Semi-automatic (UKB) dataset collected from multiple databases; the phenotypes associated with the 53 diseases in the HPO database; and combinations thereof. Figure 6 shows the ROC curves resulting from this comparison. The ROCAUC values range from 0.79 for combining Text Mined and Semi-automatic (UKB) datasets to 0.62 for only the Text Mined dataset.

### Comparison to expert-curated disease–phenotype associations

We created an expert-curated disease–phenotype association dataset to use for validation. This validation dataset consisted of 830 disease–phenotype associations for 53 diseases. To generate this dataset, we first gathered the semi-automatically curated ICD-10–HPO associations for these 53 diseases from our dataset. False positive HPO terms were filtered out and missing associations were added by an expert; 269 annotations were added. Because the HPO database contains mainly annotations to rare Mendelian diseases, most of the phenotype annotations contained in it are predicated on single gene, oligogenic, recurrent CNV or chromosome structural, disease etiology. While much of the phenotype annotation we need for common disease may be obtained from these annotations, the HPO data includes many phenotypes that are only found in the genetic syndromic disease and not in sporadic occurrences; this is discussed below. Consequently, in putting together the validation dataset, phenotypes which are not found in sporadic disease were treated as false positive unless the ICD class explicitly referred to an OMIM disease. In addition, high level terms such as HP:0002664*Neoplasm*, were excluded as being of low information content.

We used this corpus to evaluate the datasets we generated by comparing phenotype classes associated with diseases directly, using two types of evaluation, “strict” and “soft”. We called an evaluation strict if we ignored the hierarchy and semantics of phenotype ontologies and only compared whether phenotype classes matched exactly between our dataset and our benchmark. In the soft evaluation, we first propagated disease–phenotype associations over the phenotype ontology hierarchy and then evaluated on all levels of the ontology.

Our semi-automatically curated dataset covered a total of 649 disease–phenotype associations for those 53 diseases. 568/649 of the associations were true positives, 81/649 were false positives. We missed a total of 262/830 annotations (false negatives). We estimated the Precision as 0.88, Recall as 0.68 and F-score as 0.77.

**Fig. 7:**
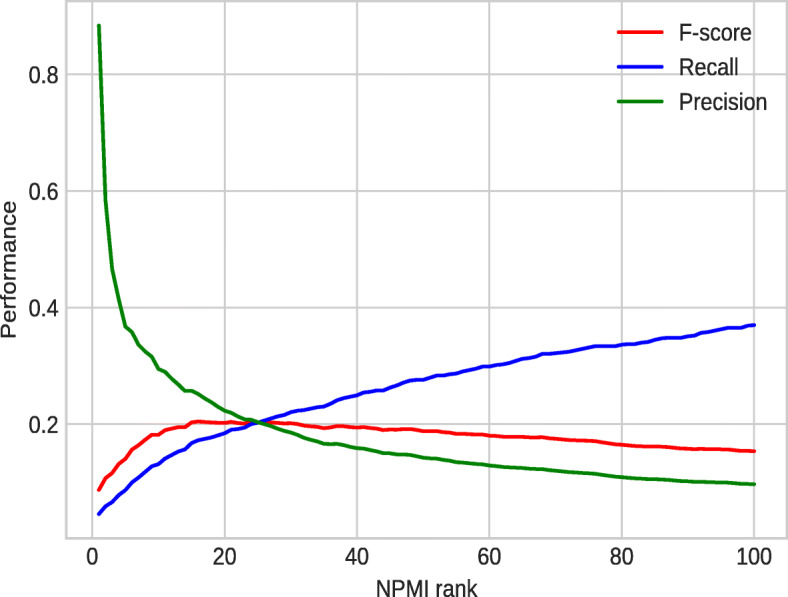
Performance analysis of text mining against the validation dataset over different NPMI ranks (strict)

Figure 7 shows the performance analysis of the text mining extracts against the validation dataset. The performance of the text mining process varied over different NPMI ranks. Max F-score value of 0.21 was achieved at NPMI rank 16.

We have a total of 3,499 disease-phenotype annotations in the validation dataset when we propagate annotations based on the PhenomeNET ontology. On the other hand, our semi-automatically curated dataset covers a total of 2,830 disease-phenotype annotations after the propagation process. In the “soft” settings, we found that 2,454/2,830 associations are true positive, 376/2,830 are false positive, and 1,045/3,499 are false negative. We estimated the Precision as 0.87, Recall as 0.70 and F-score as 0.78.

**Fig. 8:**
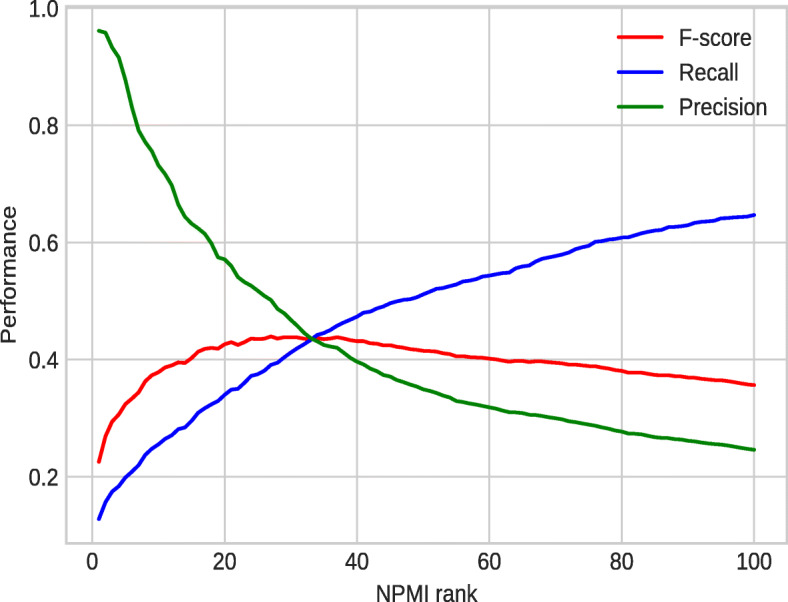
Performance analysis of text mining against the validation dataset over different NPMI ranks (soft)

Figure 8 shows the performance analysis of the text mined extracts against the validation dataset under the “soft” settings. The performance of the text mining process varies over different NPMI ranks. The best F-score is achieved at the NPMI rank of 27 as a value of 0.44.

### Coverage of the generated datasets

There are a total of 19,133 distinct ICD-10 codes. We linked 6,263 and 7,610 ICD-10 codes to their phenotypes by using text mining and the semi-automatic strategy, respectively. While we linked 4,118 ICD-10 classes to their phenotypes by both of the methods (overlap); 9,755 (51%) ICD-10 classes were linked to their phenotypes by either methods. Hence, we were unable to link 9,378 (49%) ICD-10 classes to their phenotypes. We discuss the main reasons of being unable to link these ICD-10 classes to their phenotypes in detail in the Discussion section.

### Error analysis

#### Semi-automatically curated data

We identified a total of 1,369 false positives during the semi-automatic curation of the associations from all of the 2,106 common diseases. We found that, while 963/1,369 false positives were due to the associations from existing resources, the remaining 406/1,369 false positives were due to the propagation of the annotations. 170/406 false positives are due to their lexical superclass matches in the HPO dataset and 236/406 false positives are due to their ICD-10 superclass-based annotation propagation. For example, ICD-10:C43.5*Malignant melanoma of the trunk* produced the annotation to HP:0007716*Uveal melanoma*, due to propagation from ICD-10:C43, *Malignant melanoma of skin*. We gathered the association between ICD-10:C43 and HP:0007716 from the HPO database through the mapping between OMIM:155600–ICD-10:C43 from UMLS.

Further breaking down the 963 false positives generated from the known data, we found that 12/963 false positives were from the Wikidata set, 3/963 false positives were due to the ICD-10–HPO direct mappings in UMLS, 19/963 false positives were due to incorrect associations found during the manual expert curation due to inclusion of syndromic phenotypes as discussed above, and the remaining 929/963 false positives were due to the use of the asserted disease–phenotype annotations in the HPO database. We further investigated these 929 false positives. As the diseases and phenotypes are mapped to their OMIM and HPO identifiers, respectively, to obtain ICD-10 identifiers for the OMIM diseases, we investigated the portions of the false positives introduced through OMIM–ICD-10 mappings in UMLS and Wikidata. We found that 44/929 false positives were introduced due to OMIM–ICD-10 mappings from UMLS and the remaining 885/929 false positives, which constitute the majority, were introduced due to the OMIM–ICD-10 mappings from Wikidata.

For example ICD-10:I77.1, *Stricture of artery*, is annotated to HP:0002036, *Hiatus hernia*, because Wikidata maps this ICD-10 class to OMIM:208050, *Arterial tortuosity syndrome*, which has a wide clinical phenotype spectrum among which is *Hiatal hernia*. Phenotypes that would not normally be considered a manifestation of sporadic non-syndromic arterial stricture, such as *Arachnodactyly* or *Hiatus hernia* were considered false positives. However, correct annotations to HPO were obtained directly from UMLS, which provides a correct annotation HP:0100545, *Arterial stenosis*. In general, ICD-10 to OMIM mappings through Wikidata-generated candidate HPO annotations are associated with Mendelian, syndromic disease, accounting for the high number of false positives through this route. These had to be manually removed on a case-by-case basis using expert judgement, where sporadic disease would not be expected to have these associations.

False negatives, i.e. missing annotations, were called usually when the annotation was sparse but there are clear associated phenotypes available in HPO. The causes of this are interesting. For example HP:0000979, *Purpura*, was missing from the annotation to ICD-10:M31.3*Wegener granulomatosis* [52] and HP:0025188, *Retinal vasculitis* missing from systemic ICD-10:M32.9*Lupus erythematosus* [53]. In the former case, although *Wegener granulomatosis* is in OMIM (OMIM:608710), there is no clinical synopsis and it was therefore not possible to gather annotations from the HPO database. For the latter, *Systemic lupus erythematosus*, HP:0002725 is treated as a “bundled term” phenotype in the HPO database and therefore no more granular phenotype annotations are available. There are no direct HPO annotations for *Systemic lupus erythematosis* in UMLS. We cannot provide any assurance that all of the possible missing annotations have been added to the dataset, but have provided best efforts with the resources available. We hope that users might over time request the addition of phenotypes to their diseases of interest.

#### Text mined data

For the analysis of the text mined associations, we used the extracts generated based on the NPMI rank 16 which gave us the best result on the validation dataset by using the strict evaluation (precision 0.25, recall 0.17, and F-score 0.21). We have a total of 568 ICD-10–HPO pairs in this text-mined dataset. We found that 143/568 are true positives and 425/568 are false positives. We missed a total of 687 associations (false negatives). Our manual analysis on the 425 false positives show that only a small portion of them (47/425) are false positives and the majority of them (376/425) are actually true positive associations which are not covered by our validation dataset. Our validation dataset includes only the obvious and distinguishing phenotypes of diseases. These 376 associations are the associations of the diseases with the high level of HPO classes. For example, *Malignant neoplasm of stomach, unspecified* (ICD-10:C16.9) is associated with *Neoplasm* (HP:0002664) according to our text mining extracts. This is a true positive by manual analysis but was counted as a FP since it is not covered within our validation dataset as *Neoplasm* is a high level phenotype for all malignant and benign proliferative lesions and of low information content. The false positives are mainly due to the co-mentions of associated disease concepts, or negations in the publications (X is not a Y). Some examples of such associations include *Acute myeloid leukaemia* (ICD-10:C92.0) and Chronic myelomonocytic leukemia (HP:0012325) as well as Primary open-angle glaucoma (ICD-10:H40.1) and *Angle closure glaucoma* (HP:0012109). Analysis of the 687 false negative samples showed that actually 473 of 687 pairs (69%) have been extracted from the literature but they do not rank in the top 16 based on their NPMI scores of association strength. The other missing ones are mainly due to weak or no evidence in the literature. For example, there are no publications mentioning *Marfan syndrome* (ICD-10:Q87.4) and *Decreased muscle mass* (HP:0003199); there are only 2 publications mentioning Parkinson’s disease (ICD-10:G20) and *Macrocephaly* (HP:0000256) in title or abstract together in PubMed (search was done on 15th April 2021). One of the publications is published in 2021 which is not covered by our current dataset. Therefore, there is no significant supporting evidence in the literature to infer a positive association between the given disease–phenotype pairs. Other false negatives could be due to the missing disease/phenotype synonyms. Altogether, we estimated the actual performance of the text mining method (at the NPMI rank 16) as an F-score value of 0.59, a precision of 0.92 and a recall of 0.43.

## Discussion

We have previously reported an extensive annotation of the diseases in DO based on a text mining analysis of PubMed abstracts and titles [30]. This included phenotype annotations to 6,000 common, rare and infectious diseases of which 4,768 are diseases from OMIM [29]. The under-representation of sporadic, common or complex disease in this dataset and the fact that DO is not frequently used in routine clinical recording were the motivation to develop a set of HPO annotations to terms in the much larger ICD-10 terminology. Here we have carried out a large-scale text mining analysis of PubMed using term labels, synonyms and acronyms of ICD-10 codes, and augmented this new analysis with data from three publicly available annotation sources, UMLS, Wikidata and the HPO database.

While Wikidata and the HPO database contain almost exclusively phenotypes for rare diseases found in OMIM and Orphanet, they present a source of annotation that may be exploited for common disease as explained below. A similar but more limited approach to phenotypic annotation for common disease was implemented by Sarntivijai et al. [54] using ontology-driven literature mining for two classes of disease, *Inflammatory bowel disease* and Autoimmune disease, together with their subclasses in the Experimental Factor Ontology (EFO) [55]. This produced 1,452 and 2,810 disease–phenotype pairs for inflammatory bowel disease (IBD) and autoimmune disease of which 41.6% candidate IBD phenotype associations were deemed correct by manual review. Similar to the strategy we take here, the authors of the study removed non-informative phenotypes such as “All”, “Chronic”, or “Death” but unlike us excluded classes in HPO that were deemed to represent disease entities, using expert judgement. The authors discuss some of the problems we also encountered of annotation validation on existing datasets.

In attempting a large scale phenotypic annotation of a significant number of the disease concepts in ICD-10, we have noted several issues. In trying to semi-automatically generate this corpus of annotations, one question is the decision as to what should be considered as part of a phenotypic manifestations, what level of granularity should be used, and the reliability of existing sources of annotation such as Wikidata, the HPO database, and UMLS. The definition of a phenotype as an observable characteristic covers simple signs and symptoms, and syndromic manifestations, but operationally “phenotypes” are included in the HPO database that may occur in isolation as “diseases” such as *Diabetes* or Tetralogy of Fallot (HPO regards these as “bundled phenotypes” and are included for pragmatic reasons). The decision as to how to select our annotation strategy can therefore only be guided by the purposes for which these annotations are developed, and by the best outcome on evaluation. We believe that the inclusive approach we take provides a valid strategy as assessed by performance on disease/gene prediction from the MGI dataset.

We find that many rare and rather few common diseases are extensively and accurately annotated. In some cases this is due to the deep annotation in the OMIM/HPO databases, UMLS, and, to a lesser extent, in Wikidata. The mapping of ICD classes to HPO involves for the most part working through the intermediary mappings to OMIM given in UMLS or Wikidata. As discussed above, this often results in phenotype annotations designed to describe rare inherited diseases or syndromes and not common or sporadic diseases. Although ICD classes sometimes include rare diseases explicitly, most do not, and therefore the intention in annotating a patient to an ICD-10 class is that of noting common/sporadic disease unless rare disease is asserted in the ICD-10 class chosen. As a consequence, we expertly edited annotations from HPO to align with the sporadic/common disease implied by the ICD class, giving rise to an increased number of false positive calls. We did not edit when the ICD class explicitly included an OMIM disease. This process, while driven by expert opinion is nevertheless subjective and represents a potential weakness in our approach. The low recall versus high specificity we obtain in recovering MGI gene disease associations is a consequence of disease annotation in MGI being to OMIM diseases when we edited OMIM disease phenotype annotations to approach the less complex annotation expected of sporadic disease. Our validation approach is therefore limited by what annotation datasets are available, and in the absence of any other manually curated large disease/phenotype datasets we believe that this is the best approach currently available, while not optimal.

We attempted to evaluate how removing some of these deeply annotated diseases affected the validation and found overall small changes in evaluation performance. More specifically, we identified that there are 3 heavily annotated diseases out of 53 diseases in the validation dataset *Marfan’s disease* (ICD-10:Q87.4), hereditary hemorrhagic telangiectasis (ICD-10:I78.0), and *hereditary factor VIII deficiency* (ICD-10:D66). When we removed these 3 diseases from the evaluation, the performance of the semi-automatic curation drops from an F-score value of 0.77 to 0.73.

Regarding the coverage of the datasets generated, we were unable to link 49% (9,378 out of 19,133) ICD-10 classes to their phenotypes either by semi-automatic or text mining methods. The majority of the missing ICD-10 terms are *Diseases of the musculoskeletal system and connective tissue*; ICD-10:M00–M99 (2574 ICD-10 codes), Injury, poisoning and certain other consequences of external causes, ICD-10:S00–T88 (1297 ICD-10 codes) and *External causes of morbidity*; ICD-10:V00–Y99 covering ICD-10:X00-99 (1113 ICD-10 codes), ICD-10:W00–W99 (1060 ICD-10 codes), ICD-10:V00–V99 (909 ICD-10 codes) and ICD10:Y00–Y99 (635 ICD-10 codes). We miss linking these ICD-10 codes to their phenotypes due to several methodological issues as well as the data available in the resources (HPO, UMLS, Wikidata, PubMed). More specifically, we text mined ICD-10–phenotype associations from the PubMed abstracts only and full-text articles are not covered in this study, which potentially include more associations. Furthermore, we miss some association of diseases which have long labels (e.g. ICD-10:Z62.6, *Inappropriate parental pressure and other abnormal qualities of upbringing*; ICD-10:X44, *Accidental poisoning by and exposure to other and unspecified drugs, medicaments and biological substances*) and therefore they are very unlikely to be mentioned in titles or abstracts in full. In addition, some of the associations are missed due to their low NPMI signal based on our method (we considered associations having NPMI > 0). These missing ICD-10 codes cover mainly injuries, poisoning and infectious diseases which are not focus of HPO and the other resources used in this study. Therefore, lack of these classes is not likely to reduce the utility of the generated datasets for the purposes motivating their development, which is to link phenotypes to genetic variants and underlying molecular processes.

A well established problem is that for an instance of a disease in an individual patient all phenotypes will not necessarily be present and will evolve with time. A weakness of our annotation model is that phenotype associations are treated as a “bag of phenotypes” which lacks precision and flexibility. Future work will look at application of an Ontology of Biomedical AssociatioN (OBAN) data model to our results, which allows for the inclusion of qualification into the association between disease and phenotype [54].

## Conclusion

We used a semi-automatic and a text mining based method to create four datasets of disease–phenotype associations. The generated disease–phenotype associations are useful for completing the phenotype profiles of the diseases linked to clinical resources, and can be used to investigate gene–disease associations. All the data is publicly available at Zenodo (DOI:10.5281/zenodo.4726714) for community use.

## Supplementary Information

**Additional file 1** SPARQL queries. This file contains the three SPARQL queries used to extract ICD-10–phenotype associations and ICD-10–OMIM mappings from Wikidata.

## Data Availability

We make all data freely available at 10.5281/zenodo.4726713. We make the source code developed available from Github, https://github.com/bio-ontology-research-group/icdpheno

## Abbreviations

DO: Human Disease Ontology
EFO: Experimental Factor Ontology
HPO: Human Phenotype Ontology
IBD: Inflammatory Bowel Disease
ICD: International Classification of Diseases
MGI: Mouse Genome Informatics
MP: Mammalian Phenotype Ontology
NPMI: Normalized Pointwise Mutual Information
OBAN: Ontology of Biomedical AssociatioN
OMIM: Online Mendelian Inheritance in Man
OWL: Web Ontology Language
ROCAUC: Receiver Operating Characteristic Area Under Curve
UKB: UK Biobank
UMLS: Unified Medical Language System
