# Supplementary material for: Linking common human diseases to their phenotypes; development of a resource for human phenomics
Source: J Biomed Semantics. 2021 Aug 23;12:17. doi: 10.1186/s13326-021-00249-x (PMC8383460; doi:10.1186/s13326-021-00249-x)
Supplement: Supplementary file 1 — Additional file 1 SPARQL queries. This file contains the three SPARQL queries used to extract ICD-10–phenotype associations and ICD-10–OMIM mappings from Wikidata. [file 13326_2021_249_MOESM1_ESM.pdf]

We used the following SPARQL queries to retrieve associations for diseases (mapped to ICD-10) with their symptoms https://w.wiki/bxU

```
SELECT DISTINCT ?disease ?diseaseLabel ?omim ?doid ?icd9 ?icd10 ?symptom ?symptomLabel ?hp
WHERE {
  ?disease (wdt:P279+) wd:Q12136;
    rdfs:label ?diseaseLabel.
  FILTER((LANG(?diseaseLabel)) = "en")
  ?disease wdt:P780 ?symptom.
  ?symptom rdfs:label ?symptomLabel.
  FILTER((LANG(?symptomLabel)) = "en")
  OPTIONAL { ?disease wdt:P699 ?doid. }
  OPTIONAL { ?disease wdt:P492 ?omim. }
  OPTIONAL { ?disease wdt:P1692 ?icd9. }
  OPTIONAL { ?disease wdt:P4229 ?icd10. }
  OPTIONAL { ?symptom wdt:P3841 ?hp. }
}
```

and secondary effects https://w.wiki/bxQ (mapped to HPO) from Wikidata.

```
SELECT DISTINCT ?disease ?diseaseLabel ?omim ?doid ?icd9 ?icd10 ?symptom ?symptomLabel ?hp
WHERE {
  ?disease (wdt:P279+) wd:Q12136;
    rdfs:label ?diseaseLabel.
  FILTER((LANG(?diseaseLabel)) = "en")
  ?disease wdt:P1542 ?symptom.
  ?symptom rdfs:label ?symptomLabel.
  FILTER((LANG(?symptomLabel)) = "en")
  OPTIONAL { ?disease wdt:P699 ?doid. }
  OPTIONAL { ?disease wdt:P1692 ?icd9. }
  OPTIONAL { ?disease wdt:P4229 ?icd10. }
  OPTIONAL { ?symptom wdt:P3841 ?hp. }
}
```

We gathered the mappings from Wikidata by using the following SPARQL query https://w.wiki/bxj

```
SELECT DISTINCT ?disease ?diseaseLabel ?omim ?icd10 WHERE {
  ?disease (wdt:P279+) wd:Q12136;
    rdfs:label ?diseaseLabel.
  FILTER((LANG(?diseaseLabel)) = "en")
  ?disease wdt:P492 ?omim.
  ?disease wdt:P4229 ?icd10.
}
```
